# Log transformation of proficiency testing data on the content of genetically modified organisms in food and feed samples: is it justified?

**DOI:** 10.1007/s00216-019-02338-4

**Published:** 2019-12-21

**Authors:** Wim Broothaerts, Fernando Cordeiro, Philippe Corbisier, Piotr Robouch, Hendrik Emons

**Affiliations:** European Commission, Joint Research Centre (JRC), Geel, Belgium

**Keywords:** Proficiency test, Genetically modified organism, Normality, Logarithmic transformation, Performance assessment

## Abstract

The outcome of proficiency tests (PTs) is influenced, among others, by the evaluation procedure chosen by the PT provider. In particular for PTs on GMO testing a log-data transformation is often applied to fit skewed data distributions into a normal distribution. The study presented here has challenged this commonly applied approach. The 56 data populations from proficiency testing rounds organised since 2010 by the European Union Reference Laboratory for Genetically Modified Food and Feed (EURL GMFF) were used to investigate the assumption of a normal distribution of reported results within a PT. Statistical evaluation of the data distributions, composed of 3178 reported results, revealed that 41 of the 56 datasets showed indeed a normal distribution. For 10 datasets, the deviation from normality was not statistically significant at the raw or log scale, indicating that the normality assumption cannot be rejected. The normality of the five remaining datasets was statistically significant after log-data transformation. These datasets, however, appeared to be multimodal as a result of technical/experimental issues with the applied methods. On the basis of the real datasets analysed herein, it is concluded that the log transformation of reported data in proficiency testing rounds is often not necessary and should be cautiously applied. It is further shown that the log-data transformation, when applied to PT results, favours the positive performance scoring for overestimated results and strongly penalises underestimated results. The evaluation of the participants’ performance without prior transformation of their results may highlight rather than hide relevant underlying analytical problems and is recommended as an outcome of this study.

**Figure:**
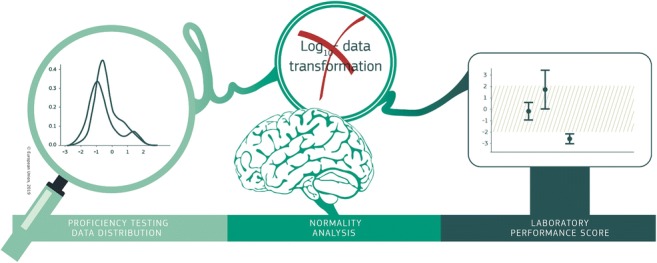
Graphical abstract

Graphical abstract

## Introduction

Proficiency tests are useful to assess the performance of laboratories for specific analytical tasks and for the identification and remediation of analytical problems [1]. For a testing laboratory, regular participation to PT rounds and obtaining satisfactory performance scores are part of the quality management system that needs to be in place in order to receive and maintain accreditation according to ISO/IEC 17025 [2]. In the field of GMO analysis, the European Union Reference Laboratory for Genetically Modified Food and Feed (EURL GMFF), hosted by the Joint Research Centre (JRC) of the European Commission, and several commercial PT providers (e.g. FAPAS, GIPSA) regularly organise PT rounds for the determination of the content of genetically modified organisms (GMOs) in food or feed test items (the PT reports issued by the EURL GMFF can be retrieved from https://gmo-crl.jrc.ec.europa.eu/Proficiency-tests.html).

Quantification of the GMO content in food or feed samples is performed in many countries in order to assess compliance to regulatory requirements regarding the authorisation of the GMO and the labelling of its presence in the product. The testing is usually done by using quantitative polymerase chain reaction (qPCR) methods applied to DNA extracted from the product. With such methods, a target DNA sequence is exponentially amplified to millions of DNA copies which can be detected fluorimetrically. The GMO content reported for a test sample is the result of applying two qPCR assays, one for the GM DNA, the other for a taxon-specific reference gene. The ratio between both amounts is expressed as GMO content. A major cause of deviation is the PCR efficiency of the assays, which is affected by the presence of inhibiting components that may remain in the DNA extracts [3]. A prerequisite for accurate GM quantification is, among others, the quality of the extracted DNA, which is influenced by the sample matrix and major processing treatments applied to the matrix [4]. The competence of testing laboratories to provide reliable data when applying such demanding analytical methods has to be demonstrated and participation in proficiency testing is an appropriate option even required when operating under ISO/IEC 17025 accreditation.

The evaluation of laboratory performance is done by PT providers in line with international general requirements [1] and statistical methods for proficiency testing [5]. Most of these statistical tests assume that a set of data is approximately normally distributed, or at least unimodal and reasonably symmetric [5]. Original datasets that appear to follow another distribution, e.g. a skewed distribution, are often logarithmic transformed to obtain a normal or near-normal distribution. Such log-data transformation is easy to perform and is included in most statistical packages. The log transformation of original data has been used, but sometimes also misused, to make data conform to normality or to reduce the variability of results in datasets that include outlying observations [6, 7].

Up to now, the reported data in all major PT schemes on the GMO content, including those organised by the EURL GMFF, have been transformed to the log_10_-scale before calculating the performance scores of the participating laboratories. Powell and Owen [8] and Thompson et al. [9] considered the positively skewed distribution of testing results on the content of GMOs collected in the frame of UK PT rounds as a mixture of normal, binomial and log-normal distributions dominated by the latter two [9]. Binomial distributions are typically seen in the case of small numbers of analysed objects, which may be present or absent as a result of sampling, or would be detected or not by an analytical method. Log-normality of repetitive results from GMO quantification methods may be caused by the successive amplification of a small number of DNA fragments in an exponential manner during qPCR. Therefore, Thompson et al. [9] recommended to log transform the reported data (expressed as a mass fraction) prior to the calculation of the performance scores (e.g. *z* scores) in order to comply with the basic assumption of “normality” set in ISO 13528 [5]. However, Feng et al. [10] demonstrated on the basis of simulated data that log-data transformation may not always be appropriate for skewed distributions and could be replaced by other approaches independent on the distribution of the data.

The present study is questioning the above-mentioned assumption of ‘log-normality’ of PT data derived from GMO quantification. It considers instead that results reported by competent participants applying validated analytical methods to quantify the measurand in a properly prepared test item would be ‘normally’ distributed.

In order to validate our assumption, the large set of PT data collected by the EURL GMFF between 2010 and 2018 was thoroughly reviewed and tested for normality in the ‘raw’ and ‘log’ scales. This data refers to a broad variety of food and feed test items, containing one or several GMOs, at GM mass fractions ranging from 0.1 to 3.8 m/m %. A total of 56 datasets (each related to one GMO per matrix) were examined. Corresponding findings and conclusions are described hereafter.

## Materials and methods

In each of the proficiency testing rounds regularly organised by the EURL GMFF over a period of 9 years (2010–2018), two test items (T1 and T2) were distributed to the participants for the quantification of one or several individual GMOs. A total of 56 datasets (including 3178 reported values) were collected and systematically re-evaluated for their departure from normality.

Before applying the normality tests, extreme outlying values or blunders were identified and excluded, as these would significantly affect the outcome of such statistical analyses. Values falling outside the range x* ± 3 s* were rejected from further calculation (where x* and s* are the robust mean and robust standard deviation of the reported results for a given PT round, calculated applying the Algorithm A method as described in ISO 13528 [5]). This procedure for outlier identification and exclusion was used only once for a dataset of a given PT round.

The Kolmogorov-Smirnov (K-S) and the Shapiro-Wilk (S-W) tests were applied to assess the ‘Goodness-of-fit’ or the departure from normality of the data for each PT round [11, 12]. The S-W test [12] is generally considered the most sensitive test for assessing departure from normality, when compared with the K-S test or with the combination of skewness and kurtosis tests [11]. No significant departure from normality is detected (the null hypothesis H_0_ is retained) when the S-W test value (*W*) is above the corresponding critical value (*W*_c_) calculated at a 95% confidence level for a given number of reported values. Statistica (TIBCO Software Inc. V13.5) was used for the statistical analysis. Statistica implements an extension to the test [13] which enables it to be applied to a large number of observations (*N* > 50).

The two normality tests (S-W and K-S) were performed for each dataset on the remaining reported values on the raw scale. No further calculation was performed when the raw dataset was proven to be normally distributed. If a significant deviation from normality was observed (*W*_raw_ ≤ *W*_c_), the normality tests were additionally applied to the log_10_-transformed data to investigate the benefit of such a data transformation with regard to the normality assumption.

## Results and discussion

### Validity of the normality assumption

The datasets collected over 9 years of proficiency testing by the EURL GMFF were obtained on a variety of food or feed test matrices and GMOs in maize, soybean and oilseed rape crops, and reported by National Reference Laboratories and official control laboratories from within and outside the EU (Table 1). The study presented here focused on the reported results expressed in GM mass per total mass, excluding the results reported in GM DNA copies per total DNA copies that were only accepted during the early years of the proficiency testing scheme (2010–2014).

**Table 1:** Normality analysis of 56 GMO proficiency testing datasets

Matrix	GMO	PT code and test item	Dataset no.	*x*_pt_ (m/m %)	*N*	*N*_SO_	*W* test value^a^		Case^c^
							*W*_raw_	*W*_log_	
100% maize	NK603 Maize	01/10-T1	1	0.1	50	1	0.93	0.97*	D
		01/10-T2	2	1.69	58	0	0.97*		A
100% maize	MON810 Maize	02/10-T1	3	0.81	63	5	0.98*		A
		02/10-T2	4	3.83	64	6	0.96	0.88	B
100% soybean	40-3-2 Soybean	01/11-T1	5	1.18	71	4	0.98*		A
		01/11-T2	6	3.52	69	3	0.97*		A
100% maize	1507 Maize	02/11-T1	7	0.30	64	3	0.96*		A
		02/11-T2	8	0.89	63	4	0.95	0.90	B
	GA21 Maize	02/11-T1	9	0.26	66	2	0.86 (0.96*)^b^	0.94	C
		02/11-T2	10	2.08	67	2	0.92	0.46	B
	MIR604 Maize	02/11-T1	11	3.38	58	5	0.94	0.76	B
		02/11-T2	12	0.89	58	5	0.96*		A
Maize and oilseed rape DNA	59122 Maize	01/12-T1	13	0.87	49	2	0.95*		A
		01/12-T2	14	3.61	48	4	0.95*		A
	GT73 Oilseed rape	01/12-T1	15	0.90	44	1	0.94	0.98*	D
		01/12-T2	16	0.39	43	2	0.97*		A
Feed-maize-soybean mix (1:1:1)	MON88017 Maize	02/12-T1	17	0.68	51	2	0.94	0.96*	D
		02/12-T2	18	1.42	51	1	0.99*		A
	40-3-2 Soybean	02/12-T1	19	1.78	63	6	0.99*		A
		02/12-T2	20	0.21	62	5	0.97*		A
Baked biscuits	MON810 Maize	01/13-T1	21	0.91	53	3	0.98*		A
		01/13-T2	22	0.36	53	3	0.97*		A
	MON863 Maize	01/13-T1	23	1.36	55	4	0.80 (0.95*)^b^	0.91	C
		01/13-T2	24	0.53	55	4	0.73 (0.98*)^b^	0.92	C
	98140 Maize	01/13-T1	25	0.33	49	4	0.98*		A
		01/13-T2	26	1.06	49	4	0.96*		A
Rice noodles with 20% soybean	356043 Soybean	02/13-T1	27	0.57	48	1	0.97*		A
		02/13-T2	28	1.35	48	1	0.96*		A
Feed-maize-soybean mix (1:1:1)	NK603 Maize	01/14-T1	29	0.60	52	1	0.82 (0.98*)^b^	0.91	C
	40-3-2 Soybean	01/14-T1	30	1.05	59	3	0.98*		A
	MON88017 Maize	01/14-T1	31	0.85	50	3	0.96*		A
	MON89788 Soybean	01/14-T1	32	0.19	50	2	0.99*		A
100% soybean	MON89788 Soybean	01/14-T2	33	0.89	52	2	0.97*		A
Chicken feed mixed with soybean	40-3-2 Soybean	02/14-T1	34	1.11	70	3	0.96	0.90	B
	40278 Maize	02/14-T1	35	1.64	48	2	0.98*		A
100% maize	40278 Maize	02/14-T2	36	0.66	56	1	0.97*		A
Rice noodles with 20% soybean	356043 Soybean	01/15-T1	37	1.34	51	2	0.98*		A
100% soybean	68416 Soybean	01/15-T2	38	0.46	49	3	0.97^a^		A
Instant soup with oilseed rape	MON88302 Oilseed rape	02/15-T1	39	1.16	44	0	0.98*		A
100% soybean	81419 Soybean	02/15-T2	40	0.99	51	3	0.99*		A
Maize Tortilla chips	1507 Maize	01/16-T1	41	0.76	73	5	0.98*		A
	MIR162 Maize	01/16-T1	42	2.60	63	1	0.99*		A
100% maize	40278 Maize	01/16-T2	43	0.63	66	4	0.97*		A
Rapeseed cake	73496 Oilseed rape	02/16-T1	44	0.50	47	3	0.95*		A
	GT73 Oilseed rape	02/16-T1	45	0.30	64	7	0.94	0.97^*^	D
100% soybean	MON89788 Soybean	02/16-T2	46	0.82	69	3	0.98*		A
Soya milk powder	DAS-44406 Soybean	01/17-T1	47	0.51	63	4	0.91	0.97*	D
100% maize	VCO-1981 Maize	01/17-T2	48	1.00	55	2	0.96*		A
Chicken feed	40-3-2 Soybean	02/17-T1	49	0.80	80	1	0.98*		A
100% soybean	40-3-2 Soybean	02/17-T2	50	0.76	83	4	0.98*		A
Maize bread	MON810 Maize	01/18-T1	51	0.47	48	5	0.98*		A
	MON89034 Maize	01/18-T1	52	0.92	46	3	0.99*		A
100% soybean	68416 Soybean	01/18-T2	53	0.42	53	0	0.97*		A
Pig feed	40-3-2 Soybean	02/18-T1	54	3.47	57	2	0.97*		A
Maize-soybean mix (1:1)	Bt11 Maize	02/18-T2	55	0.80	53	2	0.93	0.84	B
	MON87701 Soybean	02/18-T2	56	0.92	54	3	0.99*		A

*x*_*pt*_, assigned value (raw scale, expressed in m/m %); *N*, total number of reported results; *N*_SO_, number of statistical outliers

^a^Shapiro-Wilk test value (*W*), on the raw or log scale, indicated with asterisk if statistically significant at 95% (*p* < 0.05)

^b^*W* value in parentheses is without the data obtained with the biased *adh1* method

^c^Case A: *W* test value significant at raw scale, case B: *W* test value not significant at raw or log scale and *W*_raw_ > *W*_log_, case C: *W* test value not significant at raw or log scale and *W*_raw_ < *W*_log_ (but biased *adh1* method), case D: *W* test value significant at log scale (multimodal distribution)

Each analysed datasets consisted of 43 to 83 reported quantitative results, including zero to seven identified statistical outliers (see “Materials and methods”). A total of 161 statistical outliers were removed from the study (*N*_SO_ in Table 1), corresponding to between 0 and 11% of the results per dataset. Extreme outliers could have various reasons, including technical/experimental errors and obvious blunders such as the swapping of results between test items or typing and copy/paste errors, or mistakes in the preparation of the calibration standards. The identification of underlying technical/experimental issues is often difficult and complex. The origin of many of these erroneous values was identified during the follow-up support given by the EURL GMFF to the participating laboratories who obtained an unsatisfactory performance score.

After exclusion of the extreme outliers, the remaining data was statistically analysed for normality. Using the Kolmogorov-Smirnov test, which compares a distribution with a reference (normal) distribution and measures the deviation between both, all 56 investigated ‘raw’ datasets had no significant departure from normality (*p* < 0.05; data not shown). According to the Shapiro-Wilk (S-W) test, the data of 41 of the 56 ‘raw’ datasets were shown to be normally distributed with a 95% probability (case A in Table 1, for which *W*_raw_ > *W*_c_). The remaining 15 datasets, having a *W*_raw_ value that was not statistically significant, were further investigated on the logarithmic scale. For six of these 15 datasets, an increased deviation from normality in the log_10_-data transformed scale compared with the raw scale was observed (*W*_log_ < *W*_raw_ ≤ *W*_c_; case B in Table 1) and, therefore, this data transformation is not encouraged. The log_10_-data transformation improved the closeness to normality for the remaining nine datasets, i.e. the *W* test value was higher after log_10_-data transformation than on the raw scale. For four of these datasets, however, also the transformed data distributions were statistically not close to a normal distribution at a 95% probability (*p* < 0.05) and, consequently, the null hypothesis could not be rejected (case C). It is concluded, on the basis of the statistical analysis, that no log_10_-data transformation is required for 91% of the investigated datasets (51 out of 56).

The four datasets for which the log transformation resulted in a higher, but not statistically significant, *W* value (case C) were all derived from measurements on one of three PT materials containing the maize GM events NK603, GA21 or MON863. Broothaerts et al. [14] showed that the measurement method targeting *adh1* (*adh1*-70 basepairs [bp]) as taxon-specific reference sequence for the quantification of these three maize GMOs can be biased by a primer mismatch. When the reported data obtained using the unreliable *adh1*-70bp method was removed from these datasets, all of them became significant at the raw scale (Table 1, footnote b). This indicates that the analytical problem caused by the use of a biased method affected the normality of the distributions. It is not a good practice to hide this by data transformation. Instead, the performance evaluation at the raw scale would have highlighted the problem and should be used to educate the participants not to use this method any longer.

**Fig. 1:**
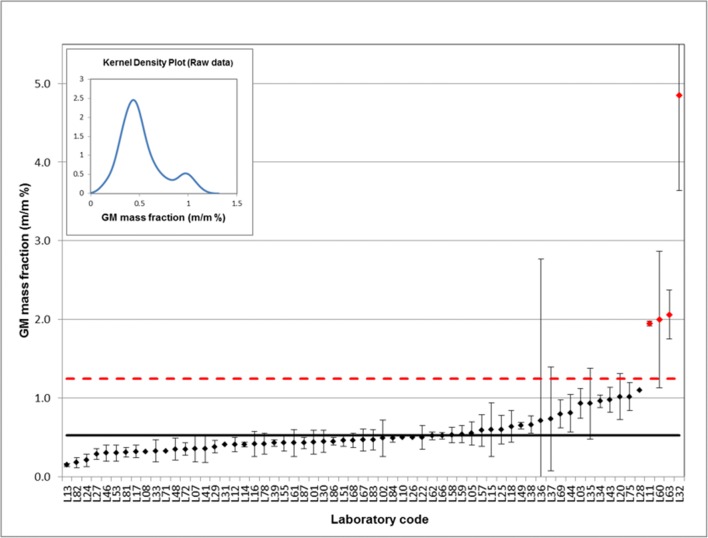
Results (and associated measurement uncertainties) reported by participants for PT 01/17-T1 (GM soybean DAS-44406) and kernel density plot. Assigned value (solid black line); statistical outliers (*N*_SO_) and outlier acceptance range (dashed line) shown in red

For the five remaining datasets, the *W* value was statistically significant only in the log domain and the null hypothesis should, in principle, be rejected (case D in Table 1). These datasets, which seemed to be log_10_-normally distributed, were further evaluated. Despite the fact that the data for each GMO are resulting from the use of the same validated measurement method (all EU enforcement laboratories are using the event-specific qPCR methods validated by the EURL GMFF, see https://gmo-crl.jrc.europa.eu/gmomethods), all five distributions appeared to be multimodal. The kernel density plot of the PT 01/17-T1 dataset (DAS-44406 soybean in soya milk powder) is presented in Fig. 1 to illustrate this observation. Such a bimodal distribution highlights some experimental/technical issues to be further investigated. The subsequent log-normal distribution suggested by the corresponding *W*_log_ value is therefore unrealistic. It simply shows that the mathematical transformation of reported results has significantly reduced the difference between the two modes, while hiding the minor mode under the major one. Similar multimodal distributions (data not shown) were observed for the remaining four ‘pseudo’ log-normally distributed datasets denoted as case D.

Multimodality of a distribution suggests that the dataset is contaminated with outlying values. Although common statistical analysis techniques for PTs assume that the data are normally distributed, ISO 13528 clearly specifies that this assumption is only valid for the underlying assumed distribution for ‘competent’ laboratories. Any ‘contamination’ of the results with erroneous values, which may lead to multimodality of a distribution as shown here, should not invalidate the basic assumption of normality [5].

The PT provider is required to demonstrate that the statistical assumptions are reasonable [1]. This demonstration may be based on the observed data and also on results from previous PT rounds [5]. As further evidence for the validity of the assumption of normality of these PT datasets, the pairwise comparable datasets that occur within the pool of 56 distributions were investigated. These were derived from the same measurand, i.e. the same GMO in the same test item matrix (used as T1 and T2), but with a different GM mass fraction. This was, e.g., the case for PT 01/10 (NK603), PT 01/12 (GT73) and PT 02/12 (MON88017), where one of the datasets of the pair was case D, but the other appeared normal on the raw scale (Table 1). This indicates that the analytical methods used for these measurements are not intrinsically leading to a deviation from normality of the resulting data. The exact reasons for the occurrence of additional modes in the data distributions may be difficult to identify. For GT73 measurements (PT 01/12 and 02/16), the deviation from normality may be related to not considering the double copy *CruA* gene used by some participants as taxon-specific reference gene [15]. As a consequence, errors were made in the conversion of the measured copy numbers into corresponding mass fractions [16]. For PT 01/17-T1 (DAS-44406 soybean), an explanation for the significant deviation from normality (observed on the raw scale) could be that the measurement method used was validated by the EURL GMFF only shortly before the PT round and that not all participants may have had the opportunity to properly implement the method in the laboratory. The matrix (soya milk powder) in test item T1 appeared to be quite difficult for a reliable extraction of good quality DNA for PCR analysis. Several participants reported to have obtained different results for this test item when using different DNA extraction methods, an observation that has already been reported before [17]. The issue was discussed during a training workshop on DNA extraction from food and feed that was organised by the EURL GMFF in 2017. All these examples suggest that technical or experimental issues may be the cause for the multimodal data distributions deviating from normality and such evidences should be taken seriously by the PT provider.

### Effect of log_10_-data transformation on the performance assessment of participants

The performance of laboratories having participated to EURL GMFF proficiency testing rounds was assessed using *z* scores, where raw or log_10_-transformed reported results (*x* or log(*x*)) have been compared with the respective assigned values (*x*_pt_ or log_10_(*x*_pt_)) and normalised with the corresponding standard deviation for performance assessment (*σ*_pt,raw_ or *σ*_pt,log_). These performance scores are expressed as:

1 $$ {z}_{\mathrm{raw}}=\raisebox{1ex}{$\left(x-{x}_{\mathrm{pt}}\right)$}\!\left/ \!\raisebox{-1ex}{${\sigma}_{\mathrm{pt},\mathrm{raw}}$}\right. $$

2 $$ {z}_{\mathrm{log}}=\raisebox{1ex}{$\left[\log (x)-\log \left({x}_{\mathrm{pt}}\right)\right]$}\!\left/ \!\raisebox{-1ex}{${\sigma}_{\mathrm{pt},\log }$}\right. $$

Replacing the *x* derived from Eq. 1 into Eq. 2, one gets:

$$ {z}_{\mathrm{log}}=\raisebox{1ex}{$\left[\log \left({x}_{\mathrm{pt}}+{z}_{\mathrm{raw}}.{\sigma}_{\mathrm{pt},\mathrm{raw}}\right)-\log \left({x}_{\mathrm{pt}}\right)\right]$}\!\left/ \!\raisebox{-1ex}{${\sigma}_{\mathrm{pt},\log }$}\right. $$

3 $$ =\log \left(1+\frac{\sigma_{\mathrm{pt},\mathrm{raw}}}{x_{\mathrm{pt}}}{z}_{\mathrm{raw}}\right)/{\sigma}_{\mathrm{pt},\log } $$

Equation 3 could be further simplified by replacing the ratio $\frac{\sigma_{\mathrm{pt},\mathrm{raw}}}{x_{\mathrm{pt}}}$ by the corresponding relative standard deviation for performance assessment (*σ*_pt,raw,rel_). Equation 3 becomes then independent from any assigned value:

4 $$ \kern0.5em {z}_{\mathrm{log}}=\log \left(1+{\sigma}_{\mathrm{pt},\mathrm{raw},\mathrm{rel}}.{z}_{\mathrm{raw}}\right)/{\sigma}_{\mathrm{pt},\log } $$

**Fig. 2:**
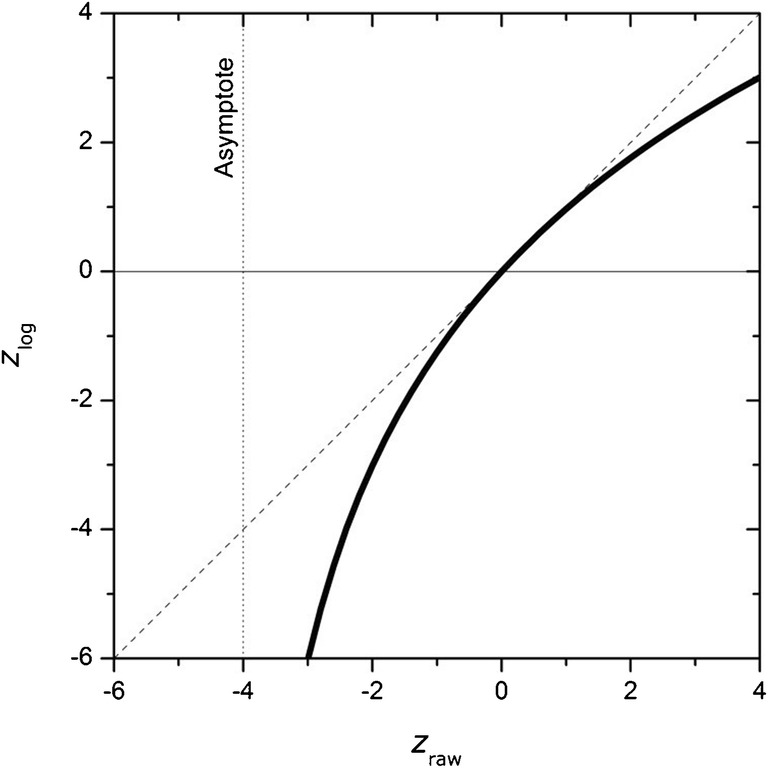
Graphical representation of *z*_log_ as a function of *z*_raw_ (Eq. 4) with *σ*_pt,raw,rel_ = 0.25 and *σ*_pt,log_ = 0.1

Equation 4 is graphically represented in Fig. 2 which shows that *z*_log_ is an increasing log function of *z*_raw*,*_ which starts with an asymptote at ‘−1/*σ*_pt, raw_’ and passes through the origin, for *z*_raw_ = 0, *z*_log_ = 0. The plotted curve is systematically below the diagonal dashed line, indicating that *z*_log_ is always lower than *z*_raw_. This implies that (i) the log_10_-data transformation improves the performance scores of laboratories having reported overestimated results (*x > x*_pt_), while (ii) it strongly decreases the performance rating for underestimated results (*x* < *x*_pt_). This is further confirmed by the following example in Table 2 where four randomly chosen sets of performance score values (*z*_raw_, *z*_log_) are calculated using *σ*_pt,raw,rel_ = 0.25 and *σ*_pt,log_ = 0.1.

**Table 2:** Relationship between performance scores (*z* scores) expressed in the raw and in the log_10_-scales

Parameter	Performance score value						
*z*_raw_	− 3	− 2	− 1.5	0	2	2.3	4
*z*_log_	− 6	− 3	− 2	0	1.8	2	3

Alternatively, Eq. 4 can be used to estimate the *σ*_pt,raw,rel_ corresponding to a *σ*_pt,log_ of 0.1, which was an assessment criterion set by the EURL GMFF in recent PT rounds. For *z*_log_ = *z*_raw_ = + 2 or − 2, two different values are obtained due to the non-symmetric nature of the log_10_ function: *σ*_pt,raw,rel_ = 0.18 and 0.29. A value of 0.25 for *σ*_pt,raw,rel_ (i.e. 25% of the *x*_pt_) could be an acceptable compromise for the evaluation of raw reported results on the content of GMOs in food or feed test items. This assessment criterion (*σ*_pt,raw,rel_ = 0.25) has been used for the evaluation of the reported results of EURL GMFF PT rounds from 2019 onwards.

## Conclusions

The assumption that data reported by competent laboratories in proficiency testing rounds on the content of GMOs in food and feed commodities is following a log-normal distribution is not generally valid. It has been demonstrated here that the majority of the 56 data distributions were normally distributed in the raw domain, thus obviating the need for log-data transformation before participant’s performance evaluation. The normality of six of the identified 15 distributions was furthermore not improving following log-data transformation, and in the remaining cases, random errors or known technical problems with the method or the test matrix may have contributed to the non-normality, as sometimes shown by the bimodality of the distributions. Thus, a data transformation approach, while seemingly appealing, would hide intrinsic technical problems rather than highlighting them.

The conclusions reported here are based on datasets from PT rounds including laboratories which are, predominantly, members of the European Network of GMO Laboratories (ENGL), who have increasingly harmonised their measurement procedures and received guidance and common training for their activities. This seems to be a reason why the meta-analysis of the PT datasets performed in this study revealed a predominantly normal distribution of the data. These datasets may, therefore, be more uniform compared with similar datasets from other PT schemes, on the basis of which a log-data transformation was recommended before performance evaluation. However, given that the methods used for GMO quantification in the EU are mostly the same for identical GM events, i.e. based on validated qPCR methods that are then applied worldwide, the common approach to log transform GMO proficiency testing data should be reconsidered also by other PT schemes. Although such transformations would often improve the performance scores of the participants, their application could hide real analytical problems that may have affected the accuracy of the reported data. Moreover, also the use of consensus values derived from the results reported by PT participants as assigned value to which participants’ performance is scored may further contribute to cover intrinsic problems.

In summary, data transformation is not justified for the reported data of the PT scheme organised by the EURL GMFF, and should be cautiously applied by other PT providers of similar PT schemes. The statistical approach described here can be exploited to PT schemes in other fields to evaluate if log transformation is justified by the reported data or not. The key message from this analysis is that a deviation from normality for a given dataset should not automatically trigger the application of a log transformation of the data. Instead, further investigations into the root causes of such deviations should be performed and their effect on the performance of the participants should be reflected in the performance scores.
